# Editorial: Sensorimotor Foundations of Social Cognition

**DOI:** 10.3389/fnhum.2022.971133

**Published:** 2022-07-08

**Authors:** Andreas K. Engel, Paul F. M. J. Verschure, Danica Kragic, Daniel Polani, Alfred O. Effenberg, Peter König

**Affiliations:** ^1^Department of Neurophysiology and Pathophysiology, University Medical Center Hamburg-Eppendorf, Hamburg, Germany; ^2^Synthetic Perceptive Emotive Cognitive Systems Laboratory, Fundació Institut de Bioenginyería de Catalunya, Barcelona, Spain; ^3^School of Electrical Engineering and Computer Science, Royal Institute of Technology, Stockholm, Sweden; ^4^Centre for Computer Science and Informatics Research, School of Computer Science, University of Hertfordshire, Hertfordshire, United Kingdom; ^5^Institute of Sports Science, Leibniz Universität Hannover, Hannover, Germany; ^6^Institute of Cognitive Science, Osnabrück University, Osnabrück, Germany

**Keywords:** social cognition, sensorimotor coupling, coordination, embodied cognition, human-robot interaction

In classical representation-oriented approaches of social cognition, agents are thought to interact with conspecifics based on their capacity to develop a “theory-of-mind”, i.e., to generate complex models of the intentions, beliefs, and personalities of their interaction partners. In this framework, the primary mode of interaction with the social environment is that of a detached observer who theorizes and produces inferences about other participants. In contrast, this Research Topic seeks to turn the spotlight on the grounding of social cognition in dynamic sensorimotor and informational coupling of agents, in human-human as well as human-robot interaction settings. According to this view, interaction dynamics hold substantial clues to the mechanism of social understanding and its disturbances (as for example observed in autism spectrum disorders). The argument is that high-level social deficits may be rooted in the impaired capacity for entraining and sustaining sensorimotor and informational coupling. Beyond novel insights into the mechanisms of functional and dysfunctional social behavior, the investigation of basic sensorimotor interaction patterns may help the development of socially competent robot technology. Tapping into the same logic, robotic agents sensitive to interpersonal sensorimotor contingencies should have an advantage over technology that does not consider this key aspect of human interaction. This Research Topic provides an interdisciplinary overview of trends and recent developments in conceptual, methodological and basic research, as well as applications of sensorimotor approaches in social cognitive science, neuroscience, and robotic research. One of the key questions is how concepts and methods from social cognitive and neuroscience transfer to human-robot interaction.

## Concepts

Several papers of this Research Topic discuss sensorimotor foundations of social cognition at the conceptual level, reviewing prevailing theories in the field and highlighting relevant experimental evidence that supports such theories. Lübbert et al. propose to extend the sensorimotor contingency theory into an action-oriented account of social cognition. The authors suggest that both informational and sensorimotor coupling between agents can support the use of action-effect contingencies in social context. The paper reviews the results of empirical studies that support the notion of socially shared sensorimotor contingencies, and discusses potential implications of this view for a better understanding of disturbed social interaction and for improvement of human-robot interactions. Vesper and Sevdalis also discuss possible functions of sensorimotor interactions in social context. They highlight three possible functions of sensorimotor communication, including the transfer of information on action intentions, the facilitation of predictions in joint action, and the stimulation of emotional experiences. A short opinion paper by Araneda Hinrichs criticizes the traditional view in social cognition research and emphasizes the usefulness of embodied and action-oriented concepts that seem more appropriate to account for social cognitive affordances. Along similar lines, an opinion piece by Rojas-Líbano and Parada argues for a key role of body-world coupling in the ontogeny of social cognition. The authors emphasize that both internal and external factors need to be considered in the modeling of social interactions by approaches from network science and by machine learning techniques. The paper by Tzafestas is a conceptual contribution reflecting upon general functions of imitation with a discussion of the nexus of the three concepts “imitation”, “association”, and “communicative function”. To this end, the long-term formation of imitation by evolution is discussed in relation to the development of basic social and communicative skills.

## Social Coordination Dynamics

This Research Topic comprises a number of behavioral studies on social coordination dynamics in a variety of different experimental paradigms. A study by Barone et al. used the perceptual crossing paradigm to develop a Turing test exploring the amount of minimally required information in terms of short-latency reciprocal sensorimotor contingencies—for human agents as well as for artificial agents. The study indicates that artificial agents should be able to generate short time reciprocal contingencies to make interactions with humans more fluid and, thus, more human-like. Using a similar paradigm, the study of Froese et al. investigated the short time emergence of sensorimotor contingencies based on haptic information by detecting the co-actor and perceptual learning of how changes in others' movements depend on changes in one's own movements, which is labeled as the “mastery of self-other contingencies”. Two related studies by Jording and coworkers address the role of gaze cues in social interaction. The first of the studies (Jording, Engemann, et al.) investigated the gaze cues that lead an observer to ascribe a social intention to the observed person's gaze. The second study (Jording, Hartz et al.) used an interactive setup for the investigation of social gaze cues, where subjects had to judge whether the other person was trying to interact with them. The results from these studies support the pivotal role of gaze in social coordination and relationship formation and show that social expectations are reflected in differential responses to gaze patterns. Trendafilov et al. used a shared task that required coordinated action by two participants, who had to move an object by jointly tilting a tablet. The authors then used transfer entropy between the participants' actions to identify leader-follower relationships. The results show that transfer entropy is sufficiently sensitive to detect leader-follower directions and, furthermore, that leader-follower relations emerge spontaneously from the interaction, without being prompted. A study by Wahn et al. asked to which degree the task setting, i.e., individual vs. joint action, can influence sensory processing. Complementary to a previous report on the effect of joint performance of a crossmodal congruency task, the authors investigated a motion-discrimination and a temporal-order judgement task. All aspects relating to single subject performance are fully compatible with previous studies. However, they did not observe an influence of joint vs. single setups on performance in the two tasks. This demonstrates that at least a part of multisensory processing is performed independently of the social setting.

## Neural and Autonomic Signatures

Grounding of social cognition in the sensorimotor and informational coupling of agents has direct implications for any investigation of the physiological substrate. Foremost, it requires measurements of the physiological signals of interacting agents. This allows relating brain activity to the types of actions performed. Recording the physiological signals from both interacting agents simultaneously (hyperscanning) also allows relating both agents' neuronal activity to each other. Compared to classical single-subject laboratory setups, the step to recording multiple interacting participants entails substantial modifications. Czeszumski et al. present a review of methods, analysis techniques and results of recent hyperscanning research. Specifically, although most available physiological recording techniques have been used in hyperscanning setups, EEG and fNIRS see the most widespread use as they allow a high degree of bodily movements of the subjects. The experimental setups often utilize rhythmic interactions and use synchronization measures to characterize the interactions between agents. Using fNIRS, the study by Su et al. investigated the synchronization of behavior and the relation to cortical activity in children and adults. They report differential activation of parietal and temporal regions during observation, execution, and joint synchronized actions. In adults, compared to children, they observed a shift of activation toward leftwards lateralization in the active conditions. Maye et al. utilized EEG recordings in a full hyperscanning setup. Importantly, they used an experimental paradigm that did not involve any external rhythmic stimulation. Under these conditions, despite quite some effort, they could not observe any direct synchronization of the two brains' activity. However, they could demonstrate brain activity in different frequency bands that correlates with objective task performance, as well as the subjective rating of task performance and collaboration of the agents. Experimental investigations are not limited to the brain's activity but also include peripheral physiological signals like heart rate variability, respiration, and electrodermal activity. Maye et al. report the surprising result that the subjective evaluation of performance by the participants can be better predicted based on such autonomic parameters compared to objective behavioral parameters. This prediction advantage of the autonomous parameters disappears in the individual settings. The coupling of autonomous parameters may be modulated by the type of relationship of the two interacting agents. Balconi and Fronda present data on the modulation of heart rate and skin conductance by exchanging gifts between two agents. Specifically, the synchronization of the heart rate increased by this experimental manipulation. These studies demonstrate that investigation of the neuronal and autonomic signatures is a fruitful field for further studies.

## Models of Social Interaction

Five articles of this Research Topic address models of social interaction, ranging from basic research on understanding and modeling of the social behavior to experimental studies on deploying and testing existing models to study human-robot interaction and coordination. Tognoli et al. investigated whether the multitudinous processes associated with social behavior abide to general principles by testing a scientific approach that tightly interweaves experimental neurobehavioral studies and mathematical models. Using the Haken-Kelso-Bunz model, its application is demonstrated in the context of social coordination in several scenarios showing, for example, that accommodating for symmetry breaking in intrinsic dynamics and coupling, multiscale generalization and adaptation are principal evolutions. Bütepage et al. studied action coordination between humans and robots, in a setting where a robot is required to learn interactive tasks from a combination of observational and kinesthetic teaching. The authors demonstrate experimentally the importance of predictive and adaptive components as well as low-level abstractions to successfully learn to imitate human behavior in interactive social tasks. Demirel et al. performed a computational analysis of sensorimotor interactions in a dual-arm robotic setup, showing that, under the common fate principle, a correlation analysis of the velocities of visual pivots is sufficient to characterize “the self” (including proximo-distal arm-joint dependencies) and to assess motor to sensory influences, and “the other” by computing clusters in the correlation dependency graph. They further show that a simple correlational analysis is not sufficient to assess the non-symmetric/directed dependencies required to infer autonomy, i.e., the ability of entities to move by themselves. Maniadakis et al. studied the temporal aspects of symbiotic human-robot interaction and explore the integration of three time-aware modules to encode past and ongoing experiences, as well as the accomplishment of goals. The integrated system is then employed to coordinate the activities of a multi-agent team. Blancas et al. investigated how impairments in prediction in young adults with autism spectrum disorder relate to their behavior during collaboration. They develop a task where participants play in interaction with a synthetic agent, and the agent's behavior changes during the game, requiring adaptation and collaboration. The results show differences between autistic and neurotypical individuals in their behavioral adaptation to the other partner but no differences in the self-reports of that collaboration.

## Author Contributions

All authors contributed to the writing of this editorial. All authors approved the submitted version.

## Conflict of Interest

The authors declare that the research was conducted in the absence of any commercial or financial relationships that could be construed as a potential conflict of interest.

## Publisher's Note

All claims expressed in this article are solely those of the authors and do not necessarily represent those of their affiliated organizations, or those of the publisher, the editors and the reviewers. Any product that may be evaluated in this article, or claim that may be made by its manufacturer, is not guaranteed or endorsed by the publisher.

